# DNA Extraction Systematics for Spectroscopic Studies

**DOI:** 10.3390/s8063624

**Published:** 2008-06-01

**Authors:** Bianca Fogazza Palma, Amanda Borges Ferrari, Renata Andrade Bitar, Maria Angélica Gargione Cardoso, Airton A. Martin, Herculano da Silva Martinho

**Affiliations:** 1 Instituto de Pesquisa & Desenvolvimento, Universidade do Vale do Paraíba, Av. Shishima Hifumi, 2911, 12244-000, São José dos Campos, São Paulo, Brazil; E-mails: bianca_palma@hotmail.com; amanda.borges@univap.br; rabc@univap.br; magcard@univap.br; amartin@univap.br; 2 Centro de Ciências Naturais e Humanas, Universidade Federal do ABC, Rua Catequese 242, 09090-400, Santo André, São Paulo, Brazil

**Keywords:** DNA, Raman spectroscopy, Cancer

## Abstract

Study of genetic material allows the comprehension the origin of the many biochemical changes that follow diseases, like cancer, promoting the development of early preventive inquiry and more efficient individual treatments. Raman spectroscopy can be an important tool in DNA study, since it allows probe molecular vibrations of genetic material in a fast way. The present work established a systematic way for extract DNA in suitable concentrations and structural integrity allowing studies by Raman spectroscopy or other spectroscopic technique, including bio-analytical sensors for probing genetic alterations.

## 1. Introduction

Diseases are always associated to biochemical changes. The goal of the frontier research in biomedical field is probe in real time and in a minimally invasive way these changes in a starting stage. This goal could be attached employing spectroscopic techniques as Raman, FTIR, fluorescence, and others to detect these changes. Among these, Raman spectroscopy had special significance since it permits analysis without prior sample preparation and in real time [1]. Recently, applications of this technique to diagnosis of diseases (Optical Biopsy) had shown great advances [2]. It had been shown to be able to differentiate several breast cancer subtypes, skin diseases, degenerative states of tendons, and other pathologic states [3].

One special characteristic present in all these pathologies is the genetic alteration. Consequently, it is usual inquire about the spectral contribution of nucleic acids (DNA and RNA) bands to the overall Raman spectra of a tissue as well as their variation with pathologic state. The development of special bio-analytic sensors for probing genetic alterations could be a direct application of this knowledge.

This work concerns the attainment of a DNA extraction methodology from human tissues that could be suitable for spectroscopic applications (mainly Raman measurements). The objective is finding a methodology providing larger yield, low concentration of expurious specimens, and guarantee of biomolecule structural integrity.

## 2. Materials and Methods

DNA was extracted from four pathological (infiltrating duct carcinoma not otherwise specified -CDI/NOS) human female breast tissue samples. Each sample was macerated and portions of 25 mg were used for the extraction procedure.

Three different DNA extraction methodologies were tested (i) DNeasy Tissue Kit (Qiagen®); (ii) ChargeSwitch gDNA Mini Tissue Kit (Invitrogen®); and (iii) in-house Phenol-Cl protocol by Sambrook *et al* [4].

Following the DNeasy protocol, samples were lysed using buffer with proteinase K and incubated in water-bath at 55°C for 3-4 hours. After that, the DNA was purified within a silica-gel-membrane spin column. For the ChargeSwitch protocol, samples were also lysed using buffer with proteinase K and incubated as described. After that the DNA purification was made with magnetic beads. In the Sambrook's protocol were used a buffer with proteinase K and SDS incubated at 60°C for 3-4 hours, followed by purification with organic solvents Phenol only and with Chloroform for at least 3 times. Preceding the purification step in all protocols, the samples were treated with Rnase A.

Extracted DNA samples quality and concentration were evaluated using UV absorption spectroscopy, agar gel electrophoresis and Raman spectroscopy.

### 2.1 UV absorption spectroscopy

UV absorption spectroscopy can be used to qualitative and quantitative characterization of biomolecules, mainly nucleic acids. Nucleic acids have a characteristic and very intense band in 260 nm [5]. The absorption spectra were measured using a ND-1000 spectrophotometer (NanoDrop® Technologies Inc., USA), which is capable to measure small volumes as 1 μL of solution without using cuvettes or other sample-holders, providing sample concentration and purity grade.

The DNA and RNA sample purity grade was estimated using the 260/280 intensity band ratio. DNA samples presenting 260/280 ratio about 1.8 were considered as free of contamination. Smaller ratios indicate contamination with proteins, phenol or others interfering compounds which strongly absorb at 280 nm [5].

### 2.2. Agar gel electrophoresis

An aliquot of 1 μL of extracted DNA sample was transferred to 5 μL of 1% agar gel electrophoresis with addition of 1 μL of 10X Blue Juice (Invitrogen™). Ethidium bromide was added in 1% agar gel and the running was made in TBE buffer (Invitrogen™) at 150 Volts for 45 minutes. The digital image of the gel was captured under UV light in a transluminator (Bio-Imaging Systems).

### 2.3 Raman spectroscopy

A FT-Raman (1064 nm) spectrometer model RFS-100 (Bruker®) was used to Raman spectroscopy measurement. The measurement parameters were 2000 scans and 250 mW laser power. Two different sample-holders were tested: capillary tube for micro-hematocrit without heparin (Mícron Glass®) and optical long path cell (Bruker®). The optical long path cell is able to amplify by a factor 10 the Raman signals of liquids.

## 3. Results and Discussion

### 3.1. UV Absorption Spectroscopy

**Figure 1.**
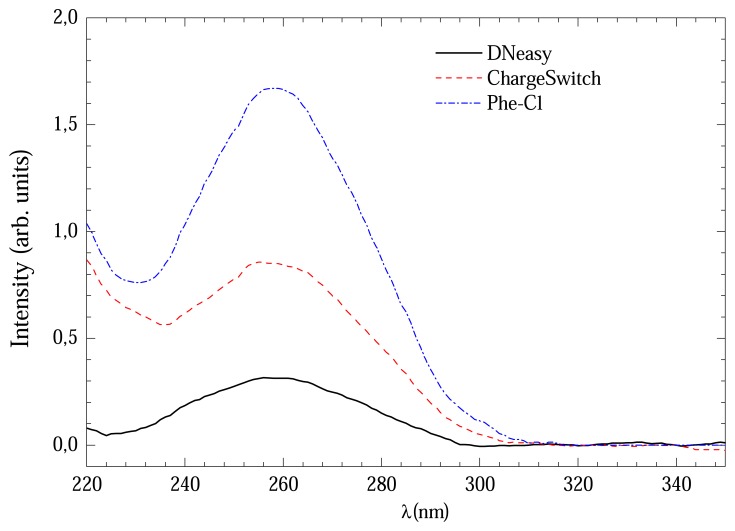
UV absorption curves of extracted DNA with DNeasy, ChargeSwitch and Phe-Cl methods with 3-4 hours of digestion.

Figure 1 shows the UV absorption curves for DNA extracted from three protocols with 3 hours of digestion time. The signal from Phenol-Cl solution was considerably more intense than others which could indicate high DNA concentration.

Table 1 shows the average (and standard deviation) concentration values obtained from four runs of each extraction method. It can be noticed that DNeasy method provided the most diluted solution with 16±8 ng/μL. However the extracted DNA sample presented a high purity grade with 260/280 ratio 1,9±0,1. The ChargeSwitch method provided solution with 85±33 ng/μL and 260/280 ratio 1,7±0,3. The Phe-Cl gave higher concentrations 93±33 ng/μL and the 260/280 ratio was 1,8±0,2.

**Figure 2.**
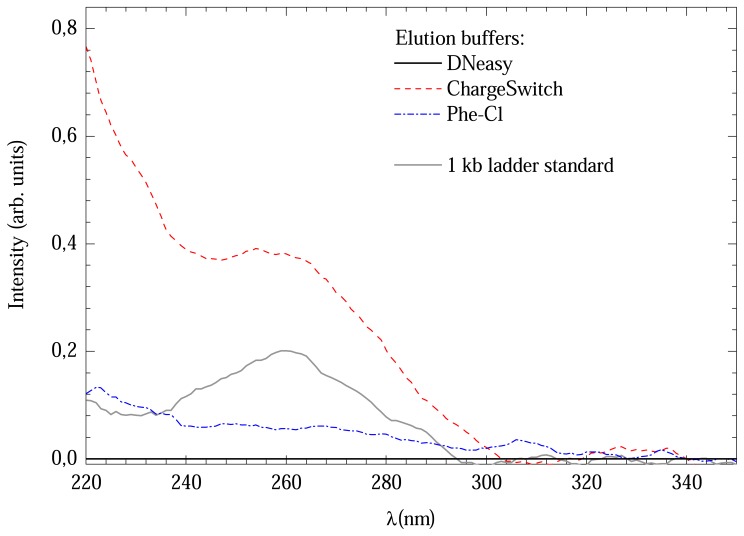
Comparison between UV absorption spectra of different elution buffers.

All methods presented good 260/280 ratios. However, the DNeasy method provided lower concentration. A possible explanation for this could be the UV absorption of the elution buffers. In order to verify this possibility, a comparison between the absorption spectra of all methods was made, which is shown in Figure 2. All, except DNeasy elution buffer, presented absorption bands near to 260 nm, where DNA absorbers. This could overestimate the DNA concentration of the solution. For comparison a 1 kb DNA ladder standard spectrum is also shown.

### 3.2. Agar gel Electrophoresis

**Figure 3.**
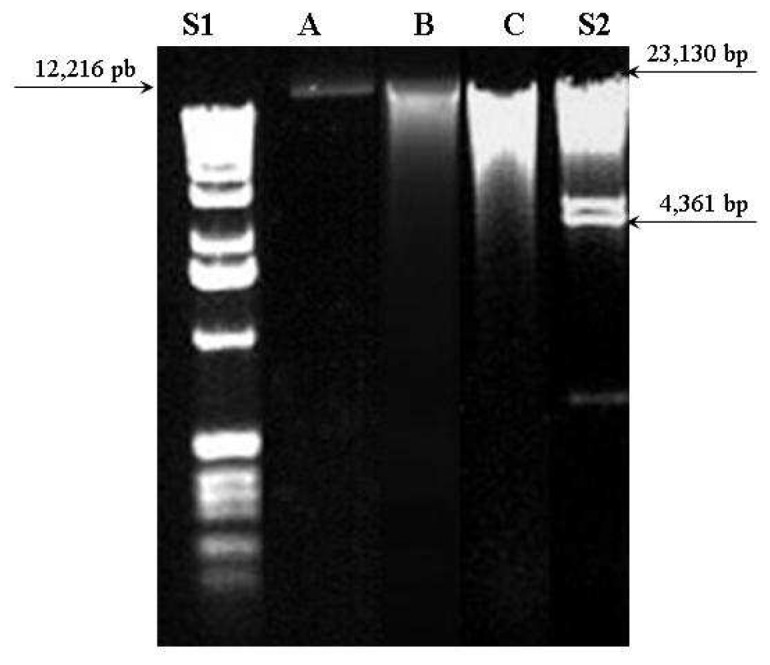
Agar gel electrophoresis 1% with ethidium bromide of extracted DNA from female breast cancer tissue. S1: 2 μL of 1Kb DNA Ladder standard sample (in base pairs-bp: 12,216; 11,198; 10,180; 9162; 8144; 7126; 6108; 5090; 4072; 3054; 2036; 1636; 1018; 506; 396; 344 and 298) A: 5 μL of DNA sample extracted by Qiagen DNeasy method; B: 5 μL of DNA sample extracted by ChargeSwitch method; C: 5 μL of DNA sample extracted by Phe-Cl method and S2: 2 μL of Hind III Fragments Standard sample (in base pairs: 23,130; 9416; 6557; 4361; 2322; 2027; 564 and 125).

The UV absorption results could be confirmed by agar gel electrophoresis. Figure 3 shows agar gel electrophoresis at 1% with ethidium bromide of DNA extracted from the three methods compared to 1 Kb DNA Ladder (S1) and λ DNA/Hind III Fragments (S2) standard samples. The standard sample S2 was prepared from λ DNA (cIind1ts857 Sam7 - *Enterobacteria phage* lambda) that was digested to completion with Hind III. This enzyme is isolated from *Haemophilus influenzae* Rd. They are suitable for sizing linear double-stranded DNA from 125 bp to 23.1 kb.

DNA from DNeasy method (A) presented a single thin band (near to 12,216 bp) which means a pure sample with non-fragmented molecule. The other two methods (B and C) presented larger and multiple bands distributed between 23,130 and 4,361 bp. This finding indicates contamination and possible DNA molecule fragmentation occurred on ChargeSwitch and Phe-Cl methods.

### 3.3 Raman spectroscopy

As first step to Raman spectroscopy characterization of DNA samples, the signal of sample-holders and elution buffers were obtained.

**Figure 4.**
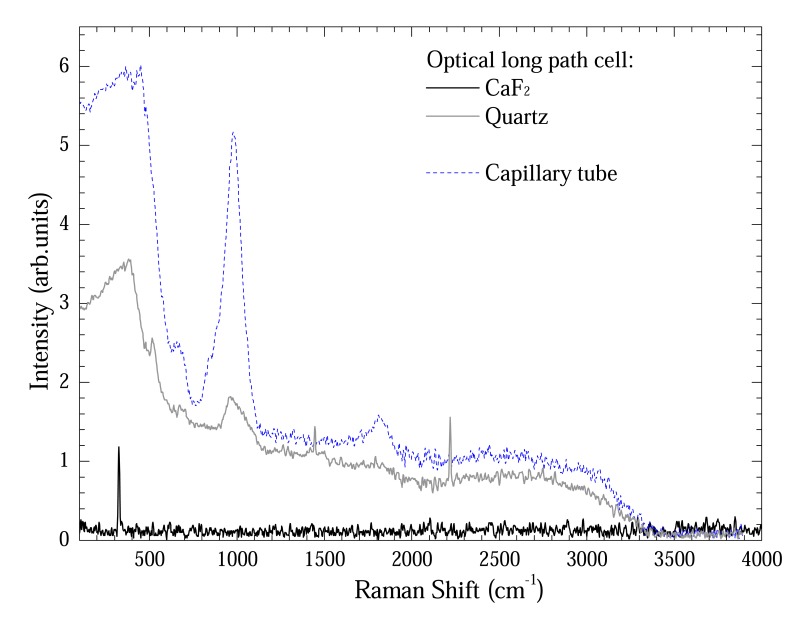
Comparison between Raman spectra from capillary tube and optical long path cell using Quartz and CaF_2_ windows.

Figure 4 shows a comparison between Raman spectra from capillary tube and optical long path cell using Quartz and CaF2 windows. Except the optical long path cell using CaF2 window, all sample-holders presented a quartz band between 100 – 1000 cm^-1^ and 1800 cm^-1^. These are Si-OH, Si-H and defect glass material vibrational modes [6]. In spite of their presence in both cases, these bands are relatively less intense for optical long path cell. Nevertheless the optical long path cell using CaF2 window presents only a band at 328 cm^-1^ [7].

The tiny bands at 1550 cm^-1^ and 2350 cm^-1^ observed at optical long path cell are air bands.

**Figure 5.**
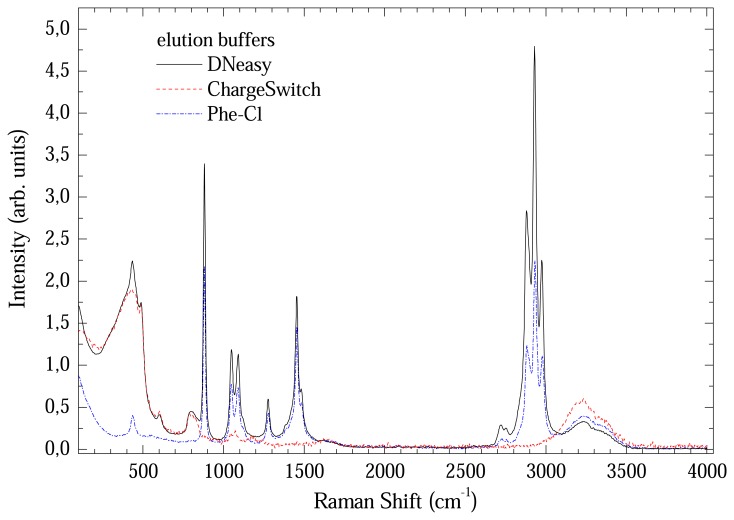
Comparison between Raman spectra of elution buffers.

Figure 5 shows a comparison between Raman spectra from the elution buffers. All presented a great number of bands up to 3500 cm^-1^. Consequently, direct elution in these buffers will completely mask the less intense DNA bands. In this case, the DNA samples needs to be eluted in a different medium. The option was use de-ionized water, which has two well known bands at 1600 and around 3200 cm^-1^.

**Figure 6.**
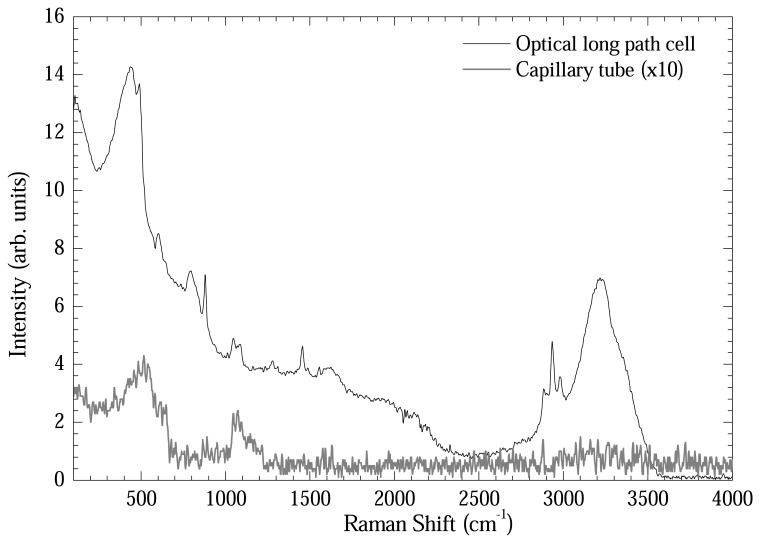
Comparison between Raman spectra from DNA extracted by DNeasy Qiagen (de-ionized water elution) method measured in optical long path cell and in capillary tube.

Figure 6 shows another comparative plot in order to test the intensification power of the optical long path cell. DNA samples extracted by Qiagen method eluted in de-ionized water were measured using the optical long path cell and the capillary tube. As could be readily seen, the Raman bands were intensified by 15-20 times using the optical long path cell.

**Figure 7.**
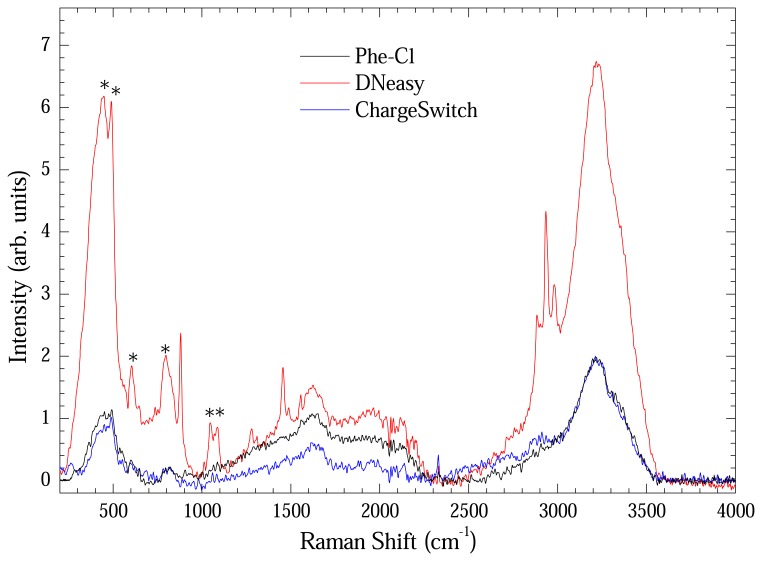
Raman spectra of extracted DNA with Phe-Cl (black line), DNeasy (red line), and ChargeSwitch (blue line) methods measured in optical long path cell. Each curve is an average of four Raman spectra. The asterisks indicate bands from the optical cell.

Figure 7 shows the Raman spectrum of extracted DNA measured using the long path cell. Each spectrum represents an average of four Raman spectra. All spectra were baseline-corrected. Vibrational modes assigned with asterisk are from the optical long path cell. Bands at 2850, 2920 and 3000 cm^-1^ remain unassigned. It is clear that the material extracted using the DNeasy method presented more intense Raman bands in spite of low concentration. It is another evidence of the preserved structural integrity of the extracted DNA with this method.

**Figure 8.**
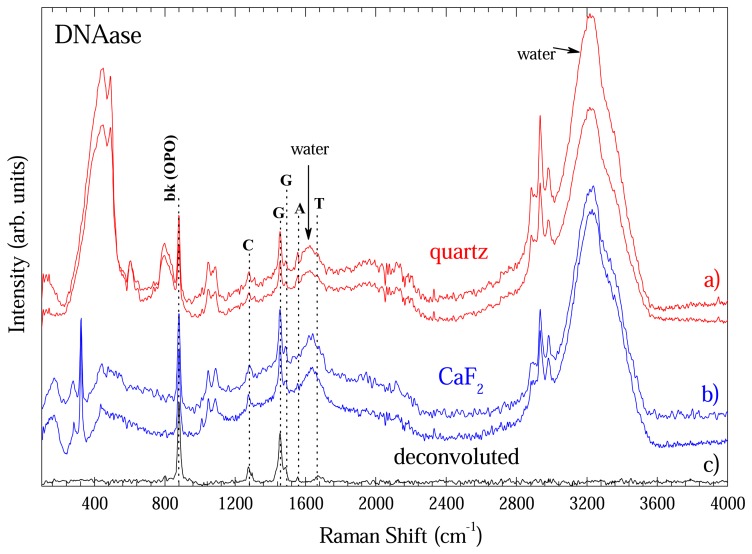
Raman spectra of DNA extracted with DNease method measured in optical long path cell. a) Data taken with quartz window. b) Data taken with CaF_2_ window. c) Average spectra after deconvolution.

Figure 8 shows some spectra extracted with DNeasy method in order to show the reproducibility of the methodology. Fig. 8a) shows two representative spectra taken with optical long path cell with quartz window. Fig. 8b) shows data taken with CaF_2_ window. This comparison is important to one become sure about the contribution going from windows. As shown in Fig.4, CaF_2_ window had only a band at 328 cm^-1^. Thus, the remaining bands are from DNA, water or optical cell. Figure 4 presented the modes of optical cell and those from water are well known what enable on to unambiguously identify the DNA bands. In fact the bands at 883; 1276; 1456/1496; 1558; and 1670 cm^-1^ could be assigned to O-P-O backbone; Cytosine (C); Guanine (G); Adenosine (A); and Thymine (T) vibrational bands of DNA and are in good accordance with ref. [8]. To clearly show the DNA bands each spectra of Fig 8a) and b) was deconvoluted by fitting the peaks to Gaussian or Lorentz lineform. The non-DNA bands fitted were subtracted from each spectra and the average of the four is on Fig. 8c).

## 4. Conclusion

The results presented in this work indicated that it is possible extract DNA from human breast tissue with suitable quality for Raman or other spectroscopic measurements, including bio-analytical sensors for probing genetic alterations. It was established that the DNeasy method with end elution in de-ionized water takes DNA with high purity grade and structural integrity.

## Figures and Tables

**Table 1.** Average and standard deviations values for concentrations and 260/280 ratio obtained with UV absorption for extracted DNA solutions.

*Method*	*Concentration (ng/μL)*	*260/280 Ratio*
DNeasy	16±8	1,9±1
ChargeSwitch	85±33	1,7±0,3
Phe-Cl	93±40	1,8±0,2
